# The Validity and Reliability of a Novel Testing Device and Six‐Way Neck‐Strength Testing Protocol for Peak Force and Rate of Force Development

**DOI:** 10.1002/ejsc.70293

**Published:** 2026-11-10

**Authors:** Molly Fownes‐Walpole, Cameron Owen, Liam Colbert, Omar Heyward, Kevin Till, Ben Jones

**Affiliations:** ^1^ Carnegie Applied Rugby Research (CARR) Carnegie School of Sport Leeds Beckett University Leeds UK; ^2^ Leeds Rhinos Rugby League Club Leeds UK; ^3^ Rugby Football League Manchester UK; ^4^ Tottenham Hotspur Football Club London UK; ^5^ Rugby Football Union Twickenham UK; ^6^ Department of Human Biology Division of Physiological Sciences Faculty of Health Sciences University of Cape Town Cape Town Western Cape South Africa; ^7^ PREM Rugby London UK; ^8^ Faculty of Health Sciences School of Behavioural and Health Sciences Australian Catholic University Brisbane Queensland Australia

**Keywords:** cervical, concussion, head acceleration events, sport, testing

## Abstract

Neck strength is hypothesised to mitigate head/neck injury and head acceleration events. Studies often only assess maximal isometric neck strength in flexion, extension, and lateral flexion and few consider the criterion and ecological validity of the devices and protocols used. This study aimed to (1): evaluate the validity and reliability of a novel strength‐testing device (LINK), and (2) determine the reliability of a practical six‐way (flexion, extension, lateral flexion, rotation) neck‐testing protocol for peak force (PF) and rate of force development (RFD). LINK validity and reliability were assessed in two parts: (A) criterion validity and inter‐/intra‐LINK reliability for PF against Kistler force platforms; and (B) criterion validity for PF and RFD during an isometric mid‐thigh pull compared with the force platforms. Intra‐tester inter‐session reliability of the neck testing protocol was determined across two sessions. Reliability was assessed using intraclass correlation coefficients (ICC). All LINKs demonstrated excellent intra‐LINK reliability. Two LINKs showed agreement with the force platforms up to 606.8 N (mean bias: 10.6 [2.8–18.4] N and 10.7 [0.6–20.8] N) and excellent inter‐LINK reliability (ICC lower bound > 0.99). One LINK was removed from further analysis due to higher mean bias (23.7 [9.6–37.8] N). The six‐way protocol demonstrated moderate‐to‐excellent reliability for PF (ICC 0.83–0.95; 95% CI between 0.53–0.99), but not for RFD (ICC 0.41–0.94; −0.30 to 0.99). Meaningful detectable changes in neck strength ranged from 38.9 to 58.0 N. This study presents a valid, reliable method for assessing neck PF across six directions.

## Highlights

- This study presents a practical, valid and reliable protocol for the assessment of maximal isometric peak force for neck extension, flexion, lateral flexion, and rotation
- Future efforts are needed to design a valid and reliable protocol for measuring neck musculature rate of force development
- This protocol can be used to further understand the impact of neck strength on head kinematics and assess the effect of neck‐training programmes

## 1 Introduction

While current evidence remains limited and inconclusive, athlete neck strength is a potentially modifiable factor that could be protective against concussion and head kinematics (Caccese et al. 2018; Fownes‐Walpole et al. 2025; Leung et al. 2024). Additionally, neck strength may remain decreased post‐concussion injury (Nutt et al. 2022), highlighting the potential for neck testing to influence injury‐prevention, training, and return‐to‐sport protocols following a concussion. Further rigorous research is needed to establish a relationship between concussion and/or head acceleration events (Ivanic et al. 2024), however, variations in neck strength assessments currently present challenges for researchers and practitioners (Peek 2022).

Neck strength has been assessed in multiple positions (e.g., seated, quadruped) using various equipment (e.g., hand‐held or fixed‐frame dynamometers, specialised equipment), and protocols (e.g., “make” or “brake”), all of which can influence measurement outcomes (Peek 2022) and make comparisons across studies difficult (Fownes‐Walpole et al. 2025). While acceptable reliability is often reported, less attention has been given to the validity of the devices and protocols used (Peek 2022). Understanding the criterion validity of a device is important to help guide training interventions based on testing data. Additionally, popular measurement equipment (e.g., VALD ForceFrame) presents challenges in practice due to its size and lack of portability. Furthermore, testing devices can be expensive and testing protocols may require additional equipment, beyond the measurement device, which can add to costs and be cumbersome to set‐up (e.g., seatbelt/harness to strap a participant in), limiting their practicality in real‐world settings. Furthermore, both the equipment and protocol should be considered to ensure the ecological validity of lab‐designed testing. For example, while testing in a four‐point position may have greater specificity to rugby union, an upright position may be more widely applicable across sports. Testing in an upright seated position has been recommended to aid comparisons prior to any further sport‐specific testing (Peek 2022).

One seated protocol using a strain gauge fixed to a squat rack demonstrated moderate‐to‐excellent intra‐tester reliability with intra‐class correlation coefficient (ICC) values between 0.83 and 0.93 (95% confidence intervals [CI] between 0.67 and 0.94) across flexion, extension, and lateral flexion (Fuller et al. 2022; Koo and Li 2016). Subsequently, this protocol has been used to assess neck strength in rugby league athletes (Leung et al. 2024; Hides et al. 2024). However, this protocol has only been used to determine maximal isometric peak force (PF) of the neck flexors, extensors, and lateral flexors. Athletes experience rotational acceleration during collisions in sport (Roe et al. 2025), which may be a better predictor of concussion injury (Tierney 2021), therefore, it is important to include measurement of neck rotators in testing and training protocols (Fownes‐Walpole et al. 2025). Furthermore, strength components beyond isometric PF are underexplored (e.g., rate of force development [RFD]) despite their potential importance in neck program designs and head impact attenuation (Fownes‐Walpole et al. 2025; Schroeder et al. 2024). A reliable, practical neck‐testing protocol capable of assessing multiple components of strength (e.g., PF and RFD) across neck flexors, extensors, lateral flexors, and rotators would be valuable.

The Remaker LINK (Version 1.2, London, UK) is a novel, affordable, and portable multi‐use strength‐testing device designed for both testing and training with multiple metrics provided to users (e.g., PF, RFD, impulse etc.). Despite its practical advantages, the validity and reliability of the LINK are unknown. Accordingly, this two‐stage study aimed to: Stage 1) assess the validity and reliability of the LINK for measuring PF and RFD; Stage 2) determine the intra‐tester, inter‐session reliability of a practical, seated, six‐way neck‐testing protocol for the determining PF and RFD.

## 2 Methods and Results

### 2.1 Study Overview

This study had two stages. Stage 1 involved the validity and reliability assessment of the LINK devices. Stage 2 involved the reliability assessment of a six‐way neck training protocol. Figure 1 demonstrates the full study protocol. Ethics approval for the study was received from Leeds Beckett University (Ref: 144020) and all participants provided informed consent.

**FIGURE 1:**
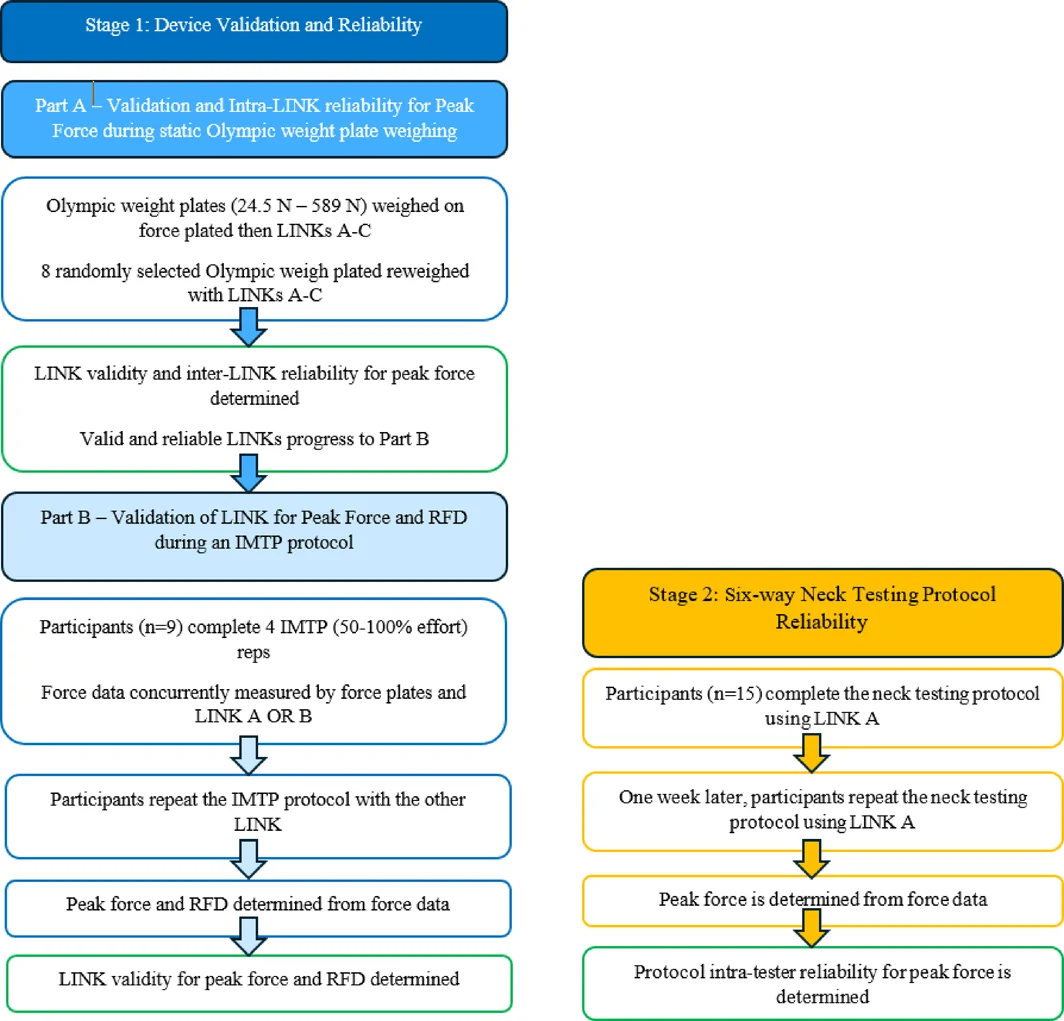
A schematic demonstrating stage 1 and stage 2 of the study design.

### 2.2 Stage 1: Device Validation and Reliability

#### 2.2.1 Study Design

Stage 1 involved two parts. In part A, three LINK devices (LINK‐A, LINK‐B, LINK‐C, 40 Hz) were assessed for criterion validity compared to in‐ground Kistler piezoelectric force platforms (Kistler Type 9253B22, Kistler, Winterthur, Switzerland), widely considered the gold‐standard criterion for force measurement. (Dos’Santos et al. 2024; Montoro‐Bombú et al. 2023; Peterson Silveira et al. 2017) Both inter‐LINK and intra‐LINK reliability for PF [N] was assessed. In part B, to assess the concurrent criterion validity of the LINKs for assessing PF and RFD [N·s‐1], LINKs were individually used during an isometric mid‐thigh pull (IMTP) test where PF was simultaneously recorded by the LINK and force platforms.

#### 2.2.2 Participants

Nine participants from a convenience sample of Leeds Beckett University members (staff and/or students) completed IMTP testing. Participants were male (*n* = 5) and female (*n* = 4) with a mean ± SD age of 27 ± 4 years, height 177.6 ± 6.3 cm, and weight 78.9 ± 8.0 kg.

#### 2.2.3 Protocol

##### 2.2.3.1 Part A

To assess the criterion validity and inter‐LINK reliability of LINKs A‐C for measuring PF, a set of Olympic plates were weighed using Kistler force platforms (Figure 2a; sampling frequency 1000 Hz) and each LINK (Figure 2b). Olympic plates were weighed with a chain and carabiner at 2.5 kg (24.5 N) intervals from 43.3 N (2.5 kg + chain) up to a combined total of 606.8 N (60 kg + chain) in a random order. To determine the intra‐LINK, test‐retest reliability, eight Olympic plates were randomly selected with and reweighed by all LINKs. Randomisation was completed using an online tool (RANDOM.ORG, Dublin, Ireland).

**FIGURE 2:**
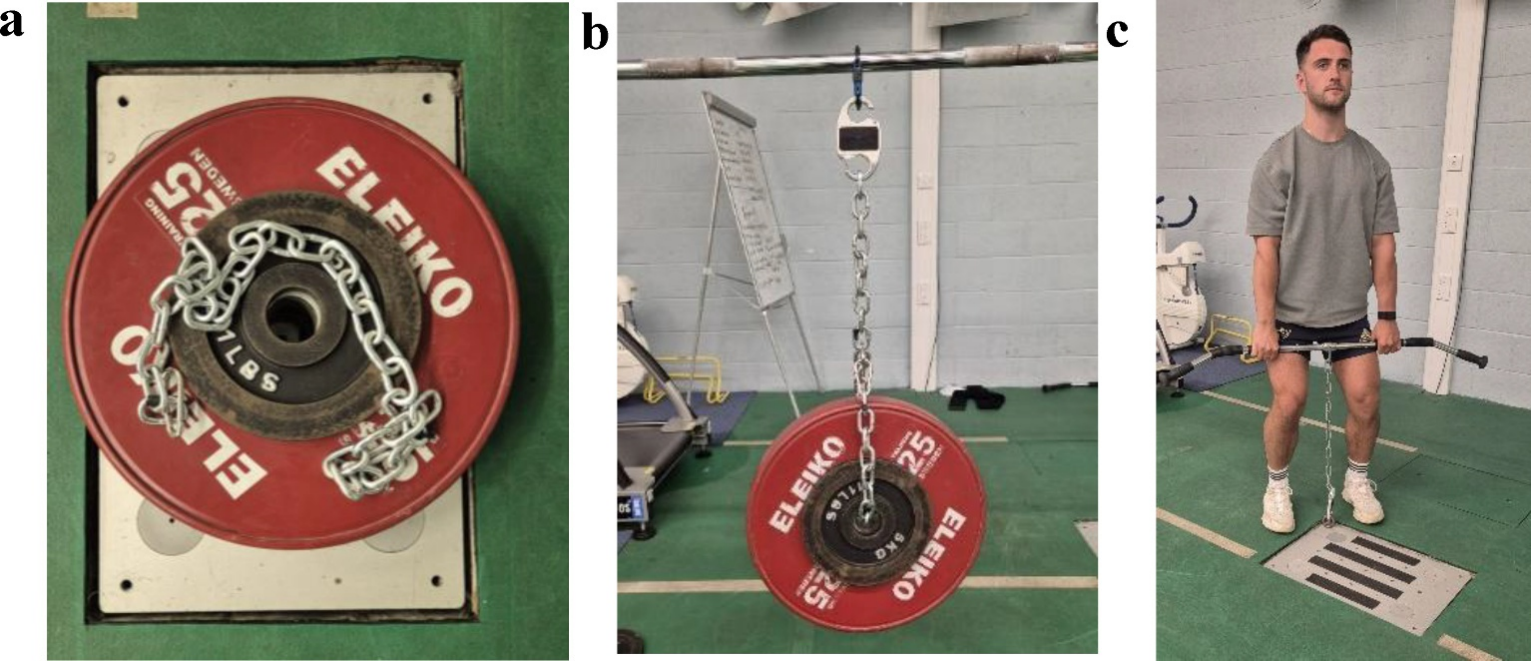
Data collection for Stage 1. Part A = the weighing of the Olympic plates using force platforms (a) and a LINK (b). Part B = the setup for the isometric mid‐thigh pull with the LINK fixed into the force platforms (c).

##### 2.2.3.2 Part B

To assess the criterion validity of the LINKs for PF and RFD concurrently to the force platforms, an IMTP test was completed (Figure 2c). Participants completed a standardised warm‐up (Supporting Information S1: Material A) before completing four IMTP attempts at perceived 50%, 70%, 90%, and 100% maximal effort with 30 s rest between reps using each LINK. The LINK was attached to the force platforms via a screwed fixture point to allow concurrent data collection. Participants stood off the plate with feet hip‐width apart, hips and knees bent, and an upright torso (Dos’Santos et al. 2017; Guppy et al. 2018). Prior to each testing session, the force platforms and LINKs were zeroed.

#### 2.2.4 Data Analysis

All data and statistical analysis took place using R Studio (2025.05.1). Vertical force platform data were collected, sign‐inverted to display positive values, and exported using the proprietary force platform software (BioWare, Version 5.3.0.7, Kistler, Winterthur, Switzerland). Force data from the LINKs was collected using the proprietary Remaker App (Version 1.17.2) and uploaded to the online Remaker portal (Remaker, London, UK). For part A, the average PF [N] recorded by the LINK across the three reps at each weight was used from the portal. For part B, raw files were provided by Remaker and max PF across any trial was used for the assessment of PF. Peak RFD was determined using a 25ms interval due to the LINK sampling frequency.

#### 2.2.5 Statistical Analysis

Bland‐Altman analyses were completed to assess the absolute and relative agreement between the devices and the force platforms for PF during parts A and B. A linear model with device as a fixed‐effect was used to determine if there was a meaningful difference between the devices (95% CIs that don't cross 0). If no meaningful difference was determined, all devices were pooled and compared to the force platforms using a Bland‐Altman plot.

Due to repeated measures in part B, a participant was added to the linear model as a random effect and a hierarchical Bland‐Altman approach was used. Proportional bias was determined and, if significant, the slope was plotted. Mean bias was estimated as the fixed‐effect intercept from the model.

Intra‐ and inter‐LINK reliability was assessed with the two‐way mixed ICC (*irr* package) (Gamer et al. 2019) in R studio. Standard error of measurement was calculated as [SEM = SD × √ (1 − ICC)] and used to determine meaningful detectable change [MDC = 1.96 × √2 × SEM]. Coefficients of variation (CV) were calculated with 95% confidence intervals [CV = (SEM/mean) × 100]. All ICCs were qualitatively interpreted based the 95% confidence intervals following the recommended interpretations: < 0.5 = “poor”, 0.5–0.75 = “moderate”, 0.75–0.9 = “good”, and > 0.9 = “excellent” (Koo and Li 2016).

### 2.3 Results: Stage 1

#### 2.3.1 Part A

LINK‐C was determined to systematically overestimate compared with LINK‐A and ‐B (Supporting Information S2: Material B). Therefore, data were not pooled and are reported by individual LINK (Figure 3). The mean bias in recorded PF between LINK‐A, ‐B, and ‐C compared to the force platforms across part A was 10.6 (2.8–18.4) N, 10.7 (0.6–20.7) N and 23.7 (9.6–37.8) N. The relative differences between LINK‐A, ‐B, and ‐C compared to the force platforms were 6.8 (−14.7 to 28.2) %, 4.4 (−3.4 to 12.3) % and 11.4 (−12.2 to 35.0) %.

**FIGURE 3:**
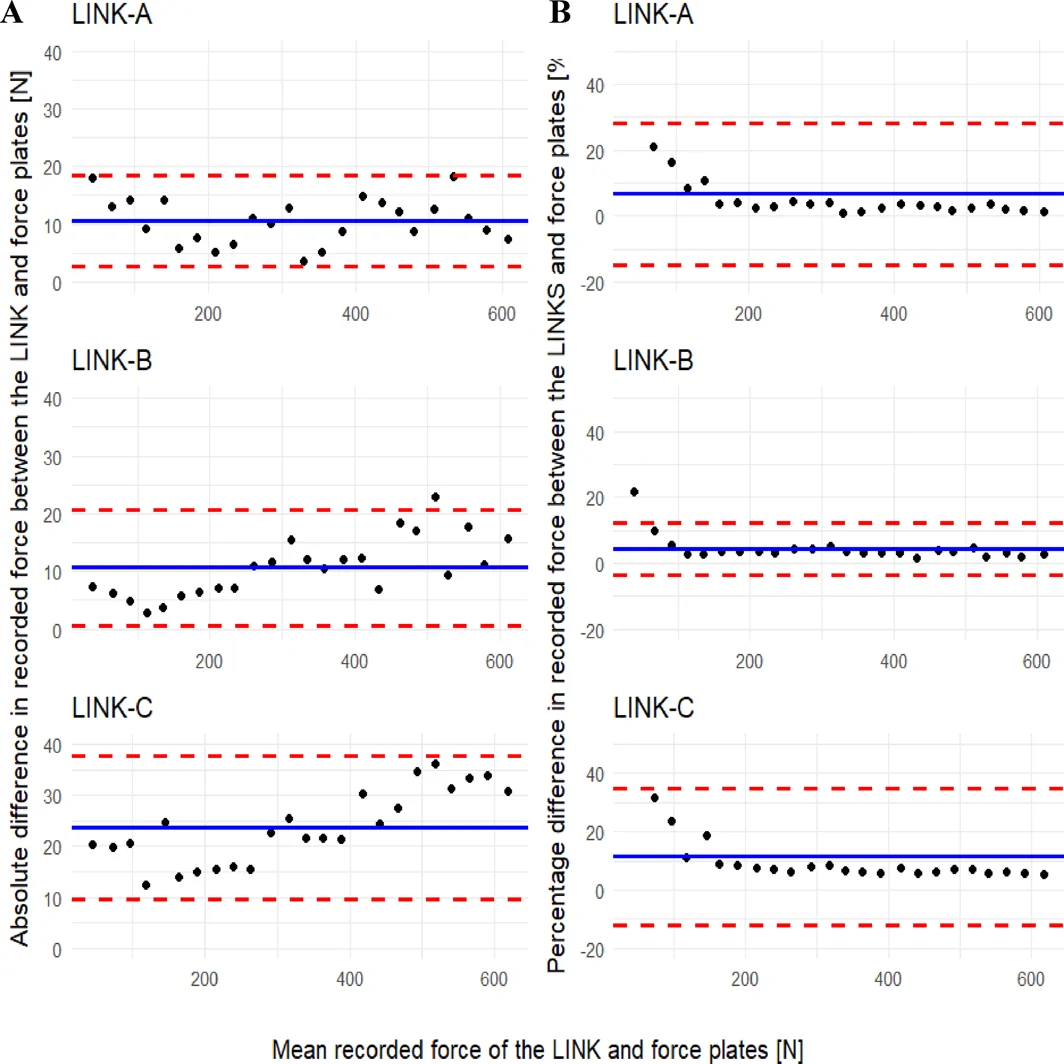
Bland‐Altman graphs demonstrating the absolute (A) and relative (B) agreement between the LINKs and force platforms. Solid blue line = mean bias. Dashed red line = limits of agreement.

The intra‐LINK test‐retest reliability and the inter‐LINK reliability measures are presented in Tables 1 and 2. Intra‐LINK reliability was excellent with mean differences ± standard deviation of −2.9 ± 5.2 N, 1.1 ± 4.0 N, and −4.1 ± 3.3 N for LINK‐A, ‐B, and ‐C. LINK‐A and ‐B demonstrated excellent inter‐link reliability with all ICC 95% CIs ≥ 0.99 and a mean difference between the two of 0.11 ± 6.55 N. When comparing LINK‐C to LINK‐A, reliability was good (ICC 95% 0.87–1.00) and compared to LINK‐B, reliability was moderate (ICC 95% 0.73–0.99), with greater mean differences (13.1 ± 7.0 N, A vs. C; 13.0 ± 4.9 N, B vs. C).

**TABLE 1:** Intra‐LINK test‐retest reliability for peak force.

LINK	MD (SD)	SEM	MDC	CV (95% CI) (%)	ICC (95% CI)	Qualitative interpretation^a^
LINK‐A	−2.86 (5.15)	3.83	10.62	1.34 (0.68–2.00)	1.00 (1.00–1.00)	Excellent
LINK‐B	1.14 (3.98)	2.66	7.37	0.93 (0.93–1.39)	1.00 (1.00–1.00)	Excellent
LINK‐C	−4.14 (3.28)	3.52	9.76	1.19 (0.61–1.78)	1.00 (0.99–1.00)	Excellent
Combined	−1.95 (4.63)	3.45	9.57	1.20 (0.86–1.54)	1.00 (1.00–1.00)	Excellent

Abbreviations: CI, confidence interval; CV, coefficient of variation; ICC, intraclass correlation coefficient; MD, mean difference; MDC, meaningful detectable change; SD, standard deviation; SEM, standard error of measurement.

^a^ Based on the bounds of the ICC.

**TABLE 2:** Inter‐LINK test‐retest reliability for peak force.

LINK	MD (SD)	SEM	MDC	CV (95% CI) (%)	ICC (95% CI)	Qualitative interpretation^a^
A v B	0.11 (6.55)	3.17	8.80	0.97 (0.69–1.24)	1.00 (1.00–1.00)	Excellent
A v C	13.12 (6.99)	7.32	20.29	2.19 (1.57–2.81)	1.00 (0.87–1.00)	Good–Excellent
B v C	13.02 (4.88)	6.86	19.01	2.05 (1.47–2.63)	1.00 (0.73–1.00)	Moderate–Excellent

Abbreviations: CI, confidence interval; CV, coefficient of variation; ICC, intraclass correlation coefficient; MD, mean difference; MDC, meaningful detectable change; SD, standard deviation; SEM, standard error of measurement.

^a^ Based on the bounds of the ICC.

#### 2.3.2 Part B

Based on the findings from part A, LINK‐C was removed from further testing. Thirty‐six IMTP attempts were recorded for LINK‐A and LINK‐B. No meaningful difference was detected between the two LINKs for assessing PF or RFD; therefore, data are reported pooled and at a LINK‐level. Figure 4 presents the agreement between the LINKs and force platforms for PF. For the pooled data, the mean bias was 69.4 (−106.7 to 272.2) N and a significant proportional bias was detected (slope = 0.065, *p* = < 0.001). At a relative level, the mean bias was 9.4 (−15.4 to 31.2) % and a significant proportional bias was detected (slope = −0.008, *p* = 0.01). For LINK‐A, the mean bias was 74.5 (−93.6 to 277.1) N and 9.5 (−0.9 to 20) %. Proportional bias was detected for the absolute peak force (slope = 0.076, *p* = < 0.001). For LINK‐B, the mean bias was 64.2 (−81 to 209) N and 9.3 (−10.5 to 29.1) %. No proportional bias was detected.

**FIGURE 4:**
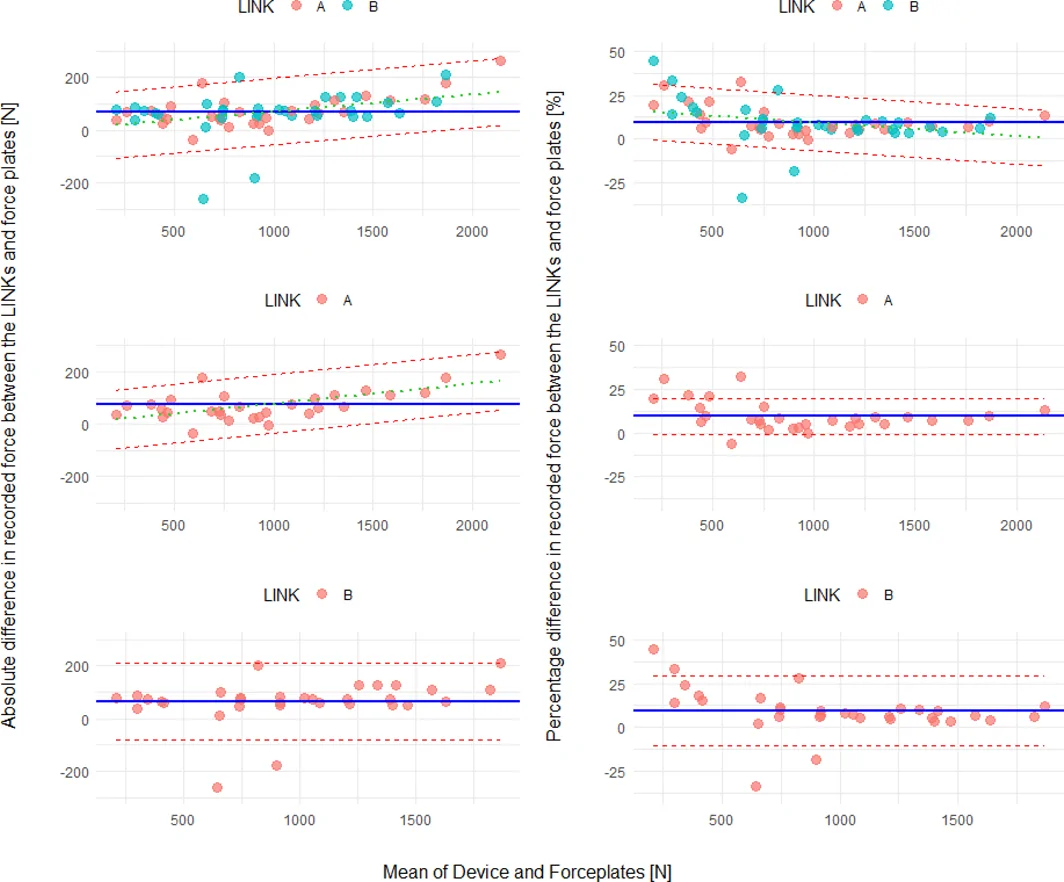
Hierarchical Bland‐Altman plots demonstrate the absolute and relative agreement between the LINKs and the force platforms for peak force. Solid blue line = mean bias. Dashed red line = limits of agreement. Dotted green lines = proportional bias.

The agreement between the LINKs and force platforms for RFD is demonstrated in Figure 5. No significant proportional bias was found for any absolute or relative values (Supporting Information S2: Material B). For the pooled data, the absolute mean bias was 276.5 (−935.8 to 1488.7) N·s‐1 and the relative mean bias was 11.7 (−24.9 to 48.2) %. For LINK‐A, these were 323.7 (−898.1 to 1545.6) N·s‐1 and 13.7 (−20.7 to 48) %. For LINK‐B, these were 227.6 (−988.7 to 1443.8) N·s‐1 and 9.6 (−29.3 to 48.5) %.

**FIGURE 5:**
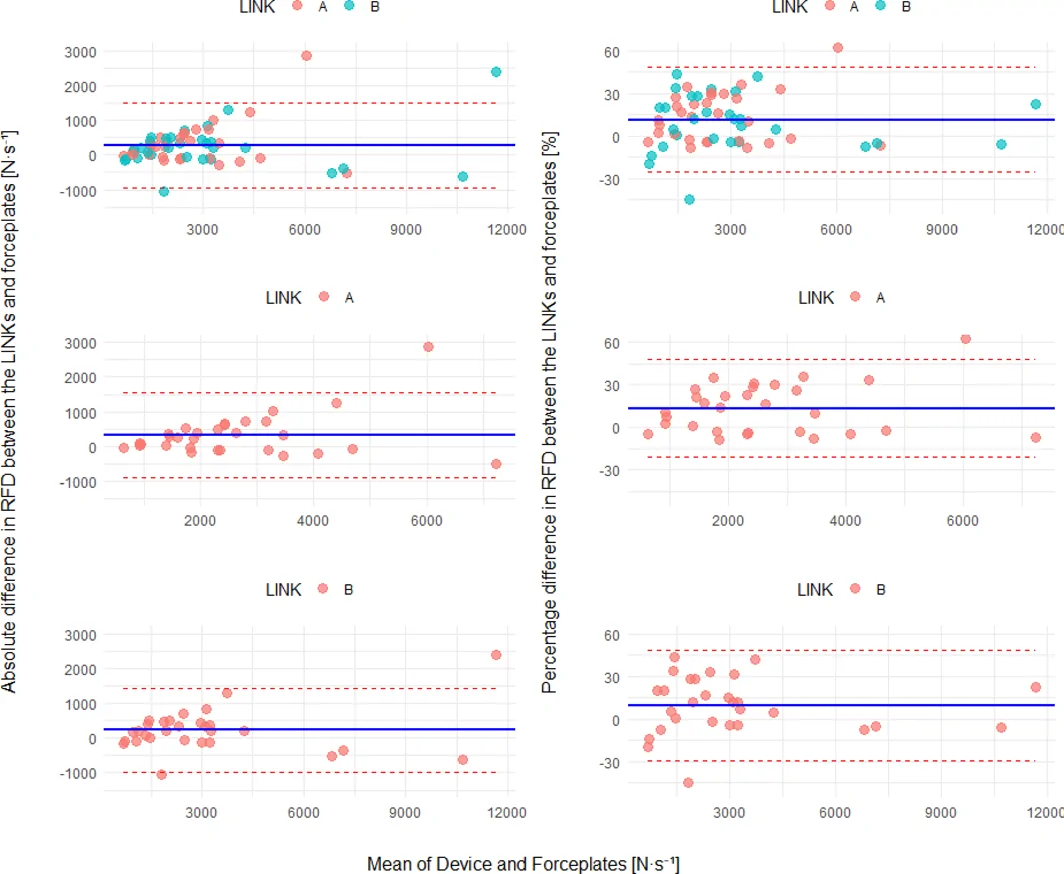
Hierarchical Bland‐Altman plots demonstrate the absolute and relative agreement between the LINKs and the force platforms for rate of force development (RFD). Solid blue line = mean bias. Dashed red line = limits of agreement.

### 2.4 Stage 2: The Reliability of a Six‐Way Neck‐Testing Protocol

#### 2.4.1 Study Design

A seated six‐way neck‐testing designed for testing in a practical setting with minimal additional equipment beyond that normally found in a gym was assessed for intra‐tester reliability across two sessions, separated by one week, with a single tester and LINK device (LINK‐A). Participants were asked to keep all physical activities the same in the 24 hours prior to each testing session. Participant consent was provided prior to testing.

#### 2.4.2 Participants

University students participating in combat or collision sports were invited to participate in the protocol reliability assessment. Participants were able to complete the study if they did not have a current shoulder/head/neck/spinal injury or suffered from neck pain. Fifteen participants from five sports volunteered (women's rugby union, *n* = 8; men's American football, *n* = 3; rugby union, *n* = 2; Thai boxing, *n* = 1; and, rugby league, *n* = 1). Participants included eight females (mean ± SD; age 20 ± 2 years, height 169.5 ± 7.4 cm, weight 84.5 ± 17.4 kg, sports participation, 6 ± 4 years), and seven males (mean ± SD; age 21 ± 2 years, height, 174.3 ± 11.1 cm, weight, 89.0 ± 23.6 kg, sports participation, 4 ± 3 years). Two female participants were excluded from analyses: one participant dropped out due to injury between testing sessions, the second participated in a rugby match prior to the second, but not first, testing session.

#### 2.4.3 Protocol

Participants completed a standardised warm‐up involving neck and shoulder exercises (Supporting Information S3: Material C). Participants were fitted with a neck harness (Iron Neck Alpha Plus, Iron Neck, Austin, Texas, USA) so that the front of the harness was placed across the frontal and parietal bones and the attachment rings aligned with the top of their ear, with the front edge just above their eyebrows. The harness was tightened by the researcher using chin, side, and top straps, and tested to ensure it was secure. Participants then completed the protocol adapted from Fuller et al. (2022) with the addition of isometric rotation (Supporting Information S3: Material C).

Briefly, participants sat upright on a weight bench, bracing themselves by gripping the bench. The LINK attached directly to the harness and via a chain to a weight rack. Participants completed a 70% familiarisation effort before completing three maximal efforts in each direction. The testing order was randomised by plane (e.g., left/right lateral flexion, flexion/extension, left/right rotation). The line of pull on the chain was horizontal and perpendicular to the attachment.

Participants were seated so the chain was tortuous to prevent movement during testing. Figure 6 demonstrates each testing position. Participants were cued to “pull away from the rack as hard and as fast as you can for three seconds” when instructed (Figure 6a,b,c). For rotational testing, to prevent side flexion, participants were instructed to “try to look over your shoulder” (Figure 6d). Each rep was recorded for five seconds using the Remaker app. Any visible movement, translation of the head on the neck, or trunk flexion resulted in a failed rep. Participants completed three reps in each direction with 10s rest between reps and 30s between directions (McBride et al. 2023). Each session took approximately 10 minutes per participant.

**FIGURE 6:**
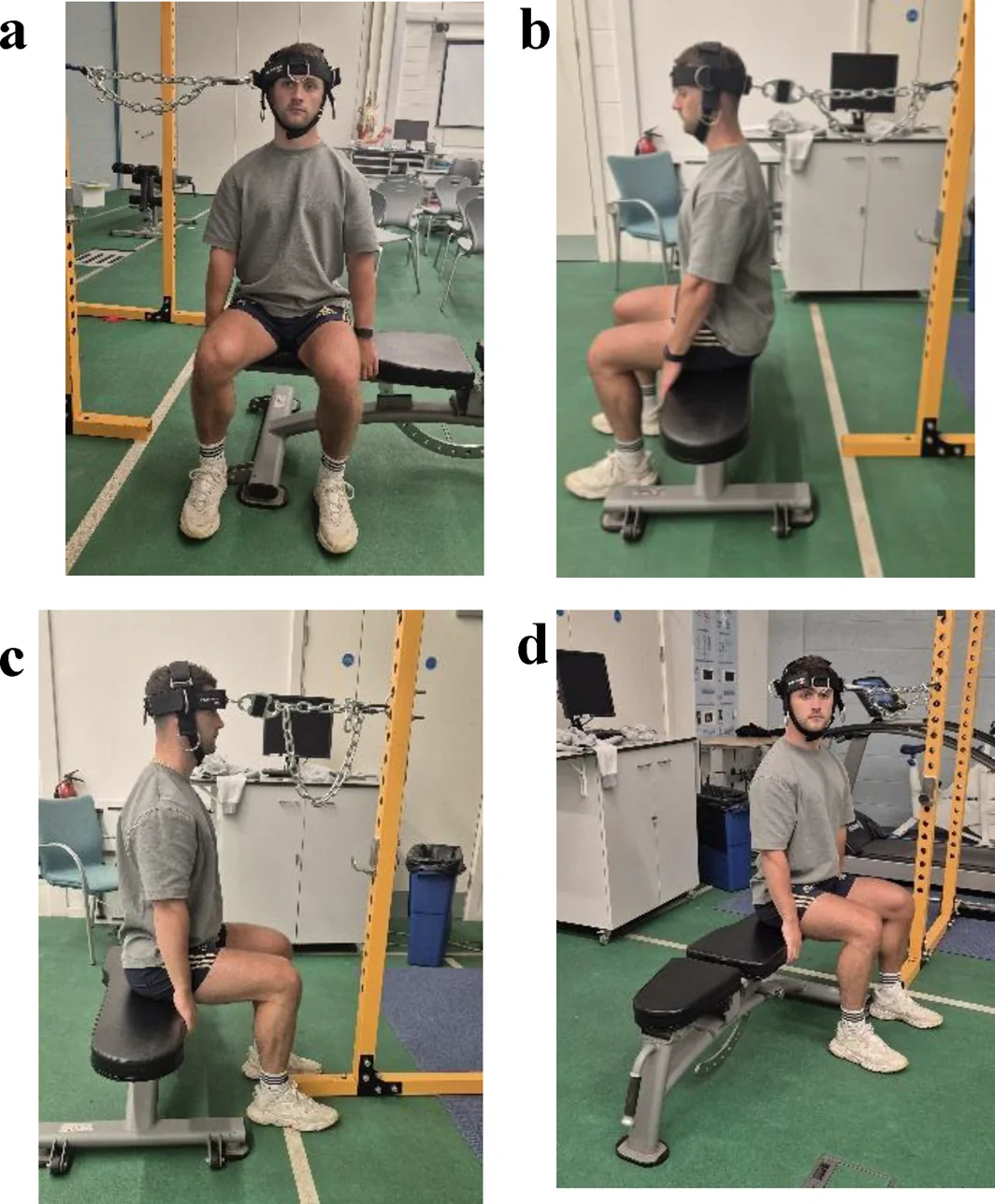
The participant set‐up for the six‐way neck testing protocol: (a) lateral flexion, (b) flexion, (c) extension, and (d) rotation.

#### 2.4.4 Data Analysis

Force data were collected using the Remaker app and raw files for each session were provided by the Remaker team. Peak forces were determined from the raw data as the absolute peak across any trial for that session in that direction, and RFD was calculated as for the IMTP. Prior to the PF and RFD analyses, the data from each trial were plotted and the graphs visually inspected to determine any reps that were not suitable for analyses (see Supporting Information S4: Material D for examples).

#### 2.4.5 Statistical Analysis

Intra‐tester protocol reliability was assessed with the two‐way mixed ICC (*irr* package) (Gamer et al. 2019). Standard error of measurement, MDC, CV and ICC were calculated and interpreted as in Stage 1.

### 2.5 Results: Stage 2

The protocol showed moderate‐to‐excellent reliability across all directions for PF but not RFD. Full reliability for PF and RFD are presented in Tables 3 and 4. No adverse effects of the protocol were reported.

**TABLE 3:** The intra‐rater inter‐session reliability of the six‐way neck testing protocol for determining peak force.

Test type	Test 1 mean (SD) [*N*]	Test 2 mean (SD) [*N*]	MD (SD)	SEM [*N*]	MDC [*N*]	CV (95% CI) (%)	ICC (95% CI)	Qualitative interpretation^a^
Flexors	153.18 (66.20)	150.10 (60.09)	−3.07 (31.02)	20.85	57.79	13.75 (8.46–19.04)	0.89 (0.67–0.96)	Moderate–excellent
Extensors	233.01 (93.38)	225.80 (86.33)	−7.21 (28.37)	19.56	54.21	8.52 (5.25–11.80)	0.95 (0.85–0.98)	Good–excellent
Right lateral flexors	184.34 (71.76)	176.13 (60.71)	−8.21 (19.27)	19.56	38.88	7.78 (4.79–10.77)	0.95 (0.86–0.99)	Good–excellent
Left lateral flexors	187.71 (71.76)	183.90 (73.07)	−3.81 (24.86)	16.79	46.54	9.04 (5.56–12.51)	0.95 (0.83–0.98)	Good–excellent
Right rotators	135.50 (49.12)	143.58 (63.82)	8.07 (30.19)	20.92	58.00	14.99 (9.23–20.76)	0.86 (0.62–0.95)	Moderate–excellent
Left rotators	140.60 (41.61)	141.57 (49.95)	0.97 (27.71)	18.59	51.52	13.17 (8.11–18.24)	0.83 (0.53–0.95)	Moderate–excellent
Composite	1034.34 (376.96)	1021.08 (375.06)	−13.81 (105.13)	70.72	196.16	6.89 (4.24–9.53)	0.96 (0.89–0.99)	Good–excellent

Abbreviations: CI, confidence interval; CV, coefficient of variation; ICC, intraclass correlation coefficient; MD, mean difference; MDC, meaningful detectable change; SD, standard deviation; SEM, standard error of measurement.

^a^ based on the bounds of the ICC.

**TABLE 4:** Intra‐rater inter‐session reliability of a six‐way neck strength protocol for determining rate of force development.

Test type	Test 1 mean (SD) [N.s‐1]	Test 2 mean (SD) [N.s‐1]	MD (SD)	SEM [N.s‐1]	MDC [N.s‐1]	CV (95% CI) (%)	ICC (95% CI)	Qualitative interpretation^a^
Flexors	747.75 (511.87)	674.96 (358.24)	−72.79 (364.13)	250.05	693.12	35.15 (21.64–48.66)	0.67 (0.22–0.89)	Poor–Good
Extensors	882.67 (431.01)	872.93 (424.34)	−9.74 (323.17)	215.54	597.45	24.55 (14.29–34.82)	0.73 (0.25–0.92)	Poor–Excellent
Right rotators	484.86 (314.58)	511.17 (458.93)	26.31 (316.15)	212.89	590.10	42.75 (25.65–59.85)	0.69 (0.21–0.90)	Poor–Excellent
Left rotators	427.19 (143.70)	404.51 (174.15)	−22.68 (176.22)	119.51	331.28	28.74 (16.14–41.34)	0.41 (−0.30–0.82)	Poor–Good
Right lateral flexors	898.63 (582.76)	768.91 (517.60)	−129.72 (147.44)	131.41	364.24	15.76 (9.17–22.35)	0.94 (0.63–0.99)	Moderate–Excellent
Left lateral flexors	981.31 (490.26)	806.13 (448.87)	−175.19 (177.66)	165.43	458.55	18.51 (10.40–26.62)	0.87 (0.30–0.97)	Poor–Excellent

Abbreviations: CI, confidence interval; CV, coefficient of variation; ICC, intraclass correlation coefficient; MD, mean difference; MDC, meaningful detectable change; SD, standard deviation; SEM, standard error of measurement.

^a^ Based on the bounds of the ICC.

## 3 Discussion

This study aimed to (1) assess the validity and reliability of the Remaker LINK for measuring PF and RFD; and (2) determine the intra‐tester inter‐session reliability of a practical, seated, six‐way neck‐testing protocol for the assessment of PF and RFD. The results of this study suggest that the LINK can validly and reliably measure PF up to 606.8 N, but individual devices may need testing before use and should not be interchanged. Additionally, this study has demonstrated that a six‐way neck‐testing protocol using common gym equipment can reliably measure neck strength PF across flexion, extension, bilateral flexion, and biaxial rotation. However, neither the LINK nor the protocol could validly or reliably assess RFD.

### 3.1 Validity and Reliability; Stage 1

Part A demonstrated that all LINKs had “excellent” intra‐LINK reliability with acceptable agreement to the force platforms for assessing PF up to 606.8 N. However, LINK‐C was identified as being meaningfully different from LINK‐A and LINK‐B with greater mean bias and poorer inter‐LINK reliability and was therefore removed from further testing. In part B, a significant proportional bias was found, indicating that as the force measured by the force platforms increased, the LINK increasingly overestimated PF. However, the percentage difference demonstrated a negative slope suggesting that the relative difference decreased with increasing force. At an individual LINK level, there were differences between devices with LINK‐A only demonstrating proportional bias for absolute PF. This, along with the part A results, indicates that where possible, the same device should be used during repeat force assessments, as has been recommended for other measurement equipment (Habets et al. 2018; Cadenas‐Sanchez et al. 2016). Additionally, the LINKs demonstrated poor validity for the assessment of RFD. A high device frequency (e.g., 1000Hz, 1ms) is recommended for the assessments of RFD during exercises such as an IMTP (Maffiuletti et al. 2016); therefore, it is likely that the LINK sampling at 40 Hz (25ms) may partially explain this finding. Therefore, the LINK cannot currently validly assess RFD compared to force platforms.

### 3.2 Validity and Reliability; Stage 2

Previously, mean neck strength has been reported within ranges of 36.5–606.1 N for soccer (Peek et al. 2020) and rugby populations (Chavarro‐Nieto et al. 2021; Geary et al. 2014; Petrie et al. 2024). However, PF in rotation and RFD are less well reported due to difficulties in measuring them (Peek 2022). Even though the LINK demonstrated poor validity for measuring RFD, protocol reliability for measuring both RFD and PF was explored as, if the protocol can reliably determine a change in RFD, this could still be useful in practice. The six‐way neck‐testing protocol demonstrated moderate‐to‐excellent reliability for assessing PF, measuring average PF between 135.5 and 225.8 N across the six directions. However, when assessing peak RFD, only isometric right lateral flexion did not demonstrate an ICC lower bound of < 0.50, suggesting poor reliability. Therefore, this neck‐testing protocol is a suitable method for the assessment of PF but not peak RFD. As mentioned, few neck testing protocols have assessed RFD (Fownes‐Walpole et al. 2025), potentially due to difficulties in determining valid and reliable devices and protocols. Studies that have determined reliable testing methods for neck RFD alongside PF have used a custom‐built neck strength testing device, which is unlikely to be practical for in real‐world settings and does not measure RFD in rotation (Almosnino et al. 2010; Nagai et al. 2020). In the present study, the LINK demonstrated poor validity for the measurement of RFD; therefore, the device and protocol should not currently be used for RFD assessment. This finding may partly be attributed to the relatively low frequency of the LINK (40 Hz). Future research should investigate whether neck RFD can be assessed using devices with increased sampling frequency. However, another study using a strain gauge also determined reliability for the assessment of PF but not RFD, despite a much higher device sample rate (1200Hz) (Burkitt et al. 2025). This study assessed only neck retraction in a prone position, which may have impacted their results.

### 3.3 Overview

The intra‐tester inter‐session reliability of the six‐way neck‐testing protocol reported in the present study is similar to the intersession reliability of other neck testing protocols (Fuller et al. 2022; McBride et al. 2023; Geary et al. 2014; Burkitt et al. 2025; Versteegh et al. 2015). A strength of this protocol is the inclusion of neck rotation. Previous neck tests have primarily focused on extensors, flexors, and lateral flexors; however, the present study has shown that neck rotation can be assessed with similar reliability. This could be important because of the rotational acceleration of the head that players experience during impacts in contact sport and the potential link between rotational acceleration and concussion (Roe et al. 2025; Tierney 2021). Versteegh et al. (2015) included rotation when using a handheld‐dynamometer. Handheld‐dynamometers are often used for strength assessments by physiotherapists and clinicians; however, they require manual application and variations in tester strength and positioning a common limitation of this method (Stark et al. 2011; Wikholm and Bohannon 1991 apr). Versteegh and colleagues overcame this challenge by having the participant self‐administer the test to avoid the influence of the strength of the tester, which can compromise the reliability (Wikholm and Bohannon 1991). While they similarly demonstrated moderate‐to‐excellent reliability between sessions, and MDC scores of 3.4–5.6 kg (33.2–55.3 N), the test is reliant on the amount of force the participant can self‐generate and standardisation between participants may be a challenge.

The present study demonstrated MDC scores between 38.9 and 58.0 N in the individual directions, suggesting changes greater than these values could have clinical significance. Studies that have investigated the effect of neck‐training interventions have demonstrated changes in neck strength beyond these thresholds in men's youth (Attwood et al. 2022) and senior (Gillies et al. 2022; Hamlin et al. 2020) rugby populations as well as in pilots (Luo et al. 2026), suggesting this protocol is capable of determining meaningful changes in neck strength. Additionally, the CV scores in the present study for PF are comparable to the limited number of studies that have assessed CV (McBride et al. 2023; Chavarro‐Nieto et al. 2021; Oxford et al. 2024). The size of the MDC scores demonstrate that the increase from baseline required to consider the change to be significant exceeds the upper bounds of the reported CV.

Limitations of this study include the lack of a familiarisation session prior to the completion of testing. This may have resulted in a learning effect between the two testing sessions; however, this was a deliberate decision as, in practice, athletes will most likely complete a baseline testing session without familiarisation. Therefore, participants were allowed a familiarisation rep prior to testing in each direction. Additionally, the protocol was designed using as little equipment beyond that found in a gym (e.g., no trunk strap) to maximise ecological validity and practicality. Because of this, it may be that the neck was not purely isolated. However, by using readily available equipment (e.g., squat rack and weights bench) it enhances the feasibility and practicality for practitioners to replicate. In practice, lack of equipment and resources has previously been reported as a barrier to implementation in other sport science research (McKay et al. 2016; Sly et al. 2022; Patterson et al. 2024; Minnig et al. 2022). Additionally, by having participants seated in an upright, unsupported position, this may better mimic scenarios where impacts are received in collision sport compared to a supine or kneeling position. Impacts in sport resulting in head/neck injuries are likely to involve coupling between the trunk and neck; therefore, although this test is not a pure assessment of neck strength, it may be more ecologically valid if prevention of these injuries is the aim. The neck testing only took place in fixed isometric positions for each direction. It is possible that variations in these positions (e.g., extension beginning from a slightly flexed or extended position) could impact the magnitude of peak force recorded. However, a neutral head position was selected because of previous reliability found in this position across flexion, extension, and lateral flexion (Fuller et al. 2022), and as it may be easier to standardise and test across larger populations. The present study cued participants to produce force as “hard and as fast as possible”. While this is the recommended approach for RFD assessment, for some populations this might be difficult and may contribute to variations in peak force outputs. In these populations, a more gradual build to peak force may be more suitable. However, concussions and head acceleration events in sport often occur during a collision where force must be generated rapidly and assessing peak force during a shorter amount of time may be more ecologically valid.

The LINK used in this protocol was determined to be valid up to 606.8 N; however, as the IMTP demonstrated, as the forces increased, the absolute agreement reduced. Therefore, consideration may be required for any participants producing peak forces beyond this threshold during neck testing. Furthermore, the protocol was unable to reliably assess peak RFD during neck testing. The relationship between neck strength and head injuries remains unclear and understanding neuromuscular changes may be important for future neck training research (Fownes‐Walpole et al. 2025); therefore, efforts to develop neck‐testing protocols that are valid and reliable for RFD should continue. The sample size may also be a limitation of this study, while we aimed to recruit more participants, only 13 participants' neck testing data could be used.

In conclusion, the present study has demonstrated that the LINK is a reliable testing device valid for assessing force up to 606.8 N. Additionally, this study presents a reliable, six‐way protocol for the assessment of neck strength in a practical setting without any adverse effects. Neck strength has seen increasing interest in athletic populations to reduce the negative consequences of head impacts; however, this association is currently unestablished and existing research is limited. Having a portable, valid and reliable means of assessing neck strength would allow practitioners and researchers to monitor the effectiveness of neck training protocols, individualise player training based on strength assessments and contribute data toward understanding the relationship between neck strength and concussion and/or head acceleration events.

## Funding

The authors have nothing to report.

## Ethics Statement

Ethical approval was granted by Leed Beckett University (Ref: 144020).

## Consent

All participants provided informed consent.

## Conflicts of Interest

The authors declare no conflicts of interest.

## Supporting information


Supporting Information S1

Supporting Information S2

Supporting Information S3

Supporting Information S4

## Data Availability

The data that support the findings of this study are available from the corresponding author upon reasonable request.
