# Supplementary material for: The Validity and Reliability of a Novel Testing Device and Six‐Way Neck‐Strength Testing Protocol for Peak Force and Rate of Force Development
Source: Eur J Sport Sci. 2026 Nov 10;26(12):e70293. doi: 10.1002/ejsc.70293 (PMC13752394; doi:10.1002/ejsc.70293)
Supplement: Supplementary file 1 [file EJSC-26-e70293-s001.docx]

Supplementary Material D – Example plots of included and excluded neck testing reps

Figure 1 a-f present examples of graphs that were included in analyses for flexion (a), extension (b), left flexion (c), right flexion (d), left rotation (e), and right rotation (f). Figure 1 g-i demonstrate examples of graphs that were excluded from analyses.

Figure 1. Examples of graphs included (a-f) and excluded (g-i) from statistical analyses

**g**

**h**

**i**

**a**

**b**

**c**

**d**

**e**

**f**
