# Supplementary material for: The Validity and Reliability of a Novel Testing Device and Six‐Way Neck‐Strength Testing Protocol for Peak Force and Rate of Force Development
Source: Eur J Sport Sci. 2026 Nov 10;26(12):e70293. doi: 10.1002/ejsc.70293 (PMC13752394; doi:10.1002/ejsc.70293)
Supplement: Supplementary file 2 [file EJSC-26-e70293-s002.docx]

Supplementary Material C - Neck Testing Protocol

**Standardised warm up**

2 x arm circles forwards and backwards

3 x nod head through full range flexion and extension

3 x move head through full range of rotation as if slowly saying no

3 x ear to shoulder through full range lateral flexion

2 x head circumduction through full range each way

3 x 3s manual resisted flexion, extension, lateral flexion 70, 80 and 90%

**Set up**

Bench perpendicular for flexion/extension

Bench in line with rack for flexion/extension/lateral flexion

Bench moved left/right of the rack for rotation (use edge of bench as marker)

Harness placed on participants head across frontal and parietal bones. The front of the harness inline with participant's brow.

Harness tightened to the point it doesn’t slip when tested

Cue for participants “I’m going to count down 3, 2, 1 and say pull pull pull, when I do I want you to pull as hard and as fast as you can”

**Player positioning (See Figure 1)**

General

- Players seated on bench
- Players brace body by using hands to grip onto bench
- Players keep feet flat on the floor
- Players maintain a neutral neck position with a slight chin tuck (cue: “like you’re making a double chin”
- Players asked to sit up straight and maintain spine and neck position throughout all tests
- Line of pull of the chain horizontal to harness attachment site
- Full tension applied to chain prior to commencement of isometric test
- Participants asked to produced maximal force as quickly as possible during each contraction
- Any visible movement away from standardised positions during the tests will count as a failed rep
- Adjust height of the chain and participant positioning if needed to ensure there is the chain is horizontal and perpendicular from the attachment at the rack to the participant

Extension

- Player seated on bench facing rack
- Bench positioned perpendicular to rack
- Player grips bench to side of legs
- Player look straight ahead
- Player instructed to maintain position throughout test and pull away from rack
- Player cue = “tension on the chain, 3, 2, 1, pull pull pull”

Flexion

- Player seated on bench facing away from rack
- Bench positioned perpendicular to rack
- Player grips bench to side of legs
- Player looks straight ahead
- Player instructed to maintain position throughout test and push away from rack
- Player cue = “tension on the chain, 3, 2, 1, pull pull pull”

Lateral flexion

- Bench parallel to rack
- Player sits side on to the rack
- Player knees in line with shoulders
- Player grips bench to side of legs
- Player looks straight ahead
- Player instructed to maintain position throughout test and pull away from rack as if they were trying to get their ear to their shoulder

**a**

**b**

**c**

**d**

Figure 1. The participant set-up for the neck testing protocol: a) lateral flexion, b) flexion, c) extension, and d) rotation

- Player cue = “tension on the chain, 3, 2, 1, pull pull pull”

Rotation (always facing into the rack)

- Player seated in same position as lateral flexion but bench is positioned a bench width left/right of the rack
- Player sits facing into the rack
- Player grips bench to side of legs
- Player tucks chin
- Player rotates head as far as they can with shoulders remaining facing forwards (approximately 45 degrees)
- Player instructed to maintain position throughout test and push into the harness, rotating away from rack as if trying to look over their shoulder
- Player cue = “tension on the chain, 3, 2, 1, pull pull pull”

Testing

- 1 submaximal 70% effort then 3 max efforts in each direction
- Make sure chain is always tort before starting – start each rep with “tension on the chain...”
