# Supplementary material for: The Validity and Reliability of a Novel Testing Device and Six‐Way Neck‐Strength Testing Protocol for Peak Force and Rate of Force Development
Source: Eur J Sport Sci. 2026 Nov 10;26(12):e70293. doi: 10.1002/ejsc.70293 (PMC13752394; doi:10.1002/ejsc.70293)
Supplement: Supplementary file 3 [file EJSC-26-e70293-s003.docx]

Supplementary Material B

This document contains the results of the testing for significant differences between devices and the presence of significant proportional bias. The following packages were used in R studio:

Lme4^1^

lmerTest^2^

1. **Part A**
   1.1. Significance testing

LINK-A vs LINK-B: Mean bias difference = -0.108, 95% CI (-2.875 - 2.659), p = 0.947

LINK-A vs LINK-C: Mean bias difference = -13.123, 95% CI (-16.0730 -10.174), p < 0.001

LINK-B vs LINK-C: Mean bias difference = -13.0158, 95% CI (-15.07616 -10.95543), p < 0.001

2. **Part B**
   2.1. Peak Force
        2.1.1. Significance testing

LINK-A vs LINK-B: Mean bias difference = -10.74, 95% CI (-43.156 - 21.676), p = 0.519

2.1.2. Proportional Bias

Pooled Absolute; slope = 0.065, 95% CI (0.27 – 0.102) p < 0.001

Pooled Relative; slope = -0.0077, 95% CI (-0.013 - -0.020) p = 0.01

LINK-A Absolute; slope = 0.076; 95% CI (0.039 – 0.113) p = 0.00027

LINK-A Relative; slope = -0.0055; 95% CI (-0.012 – 0.00056), p = 0.086

LINK-B Absolute; slope = 0.054; 95% CI (-0.012 – 0.121), p = 0.112

LINK-B Relative; slope = -0.0099, 95% CI (-0.0197 - -0.000165), p = 0.055

2.2. RFD
     2.2.1. Significant difference

LINK-A vs LINK-B: Difference in bias: -96.18, 95% CI (-413.64 - 221.29), p = 0.56

2.2.2. Proportional BIAS

Pooled Absolute: slope = 0.066, 95% CI (-0.00723 – 0.139) p = 0.0764

Pooled Relative: slope = 0, 95% CI (-0.00216 - 0.00228), p = 0.957

LINK-A Absolute: slope = 0.121, 95% CI (-0.0314 – 0.274), p = 0.115

LINK-A Relative: slope = 0.002, 95% CI (-0.00247 – 0.00583), p = 0.472

LINK-B Absolute: slope = 0.053, 95% CI (-0.349 – 0.141), p = 0.226

LINK-B Relative: slope = 0, 95% CI (-0.00304 – 0.00249), p = 0.848

References:

1.	Bates D, Mächler M, Bolker B, Walker S. Fitting Linear Mixed-Effects Models Using lme4. Journal of Statistical Software. 2015 Oct 7;67:1–48. doi:10.18637/jss.v067.i01

2.	Kuznetsova A, Brockhoff PB, Christensen RHB. lmerTest Package: Tests in Linear Mixed Effects Models. Journal of Statistical Software. 2017 Dec 6;82:1–26. doi:10.18637/jss.v082.i13
