# Supplementary material for: The Validity and Reliability of a Novel Testing Device and Six‐Way Neck‐Strength Testing Protocol for Peak Force and Rate of Force Development
Source: Eur J Sport Sci. 2026 Nov 10;26(12):e70293. doi: 10.1002/ejsc.70293 (PMC13752394; doi:10.1002/ejsc.70293)
Supplement: Supplementary file 4 [file EJSC-26-e70293-s004.docx]

Supplementary Material A – Isometric mid-thigh pull warm-up and build

Isometric Mid-Thigh Pull Warm Up

- 3 arm circles forwards and backwards
- 6 single leg lunges
- 6 bodyweight Romanian deadlift
- 6 air squats
- 4 countermovement jumps

Isometric Mid-Thigh Pull build-up (all recorded and repeated on LINK-A and LINK-B)

- IMTP at 50%
- IMTP at 70%
- IMTP at 90%
- IMTP 100%

30s rest between reps

1-2 mins rest between devices
